# Modified Rhamnogalacturonan-Rich Apple Pectin-Derived Structures: The Relation between Their Structural Characteristics and Emulsifying and Emulsion-Stabilizing Properties

**DOI:** 10.3390/foods10071586

**Published:** 2021-07-08

**Authors:** Jessika N. Humerez-Flores, Sarah H. E. Verkempinck, Clare Kyomugasho, Paula Moldenaers, Ann M. Van Loey, Marc E. Hendrickx

**Affiliations:** 1 Laboratory of Food Technology and Leuven Food Science and Nutrition Research Centre (LFoRCe), Department of Microbial and Molecular Systems (M2S), KU Leuven, Kasteelpark Arenberg 22, PB 2457, B-3001 Leuven, Belgium; sarah.verkempinck@kuleuven.be (S.H.E.V.); clare.kyomugasho@kuleuven.be (C.K.); ann.vanloey@kuleuven.be (A.M.V.L.); marceg.hendrickx@kuleuven.be (M.E.H.); 2 Department of Chemical Engineering, Soft Matter, Rheology and Technology, KU Leuven, Celestijnenlaan 200F, PB 2424, B-3001 Leuven, Belgium; paula.moldenaers@kuleuven.be

**Keywords:** apple pectin, rhamnogalacturonan-I, side chains, structural modifications, emulsion, stability

## Abstract

In the context of the increasing interest in natural food ingredients, the emulsifying and emulsion-stabilizing properties of three rhamnogalacturonan-rich apple pectin-derived samples were assessed by evaluating a range of physicochemical properties. An apple pectin (AP74) was structurally modified by a β-eliminative reaction to obtain a RG-I-rich pectin sample (AP-RG). Subsequent acid hydrolysis of AP-RG led to the generation of pectin material with partially removed side chains (in particular arabinose depleted) (AP-RG-hydrolyzed), thus exhibiting differences in rhamnose, arabinose, and galactose in comparison to AP-RG. All samples exhibited surface activity to some extent, especially under acidic conditions (pH 2.5). Furthermore, the viscosity of the samples was assessed in relation to their emulsion-stabilizing properties. In a stability study, it was observed that the non-degraded AP74 sample at pH 2.5 exhibited the best performance among all the apple pectin-derived samples evaluated. This emulsion presented relatively small oil droplets upon emulsion production and was less prone to creaming than the emulsions stabilized by the (lower molecular weight) RG-I-rich materials. The AP-RG and AP-RG-hydrolyzed samples presented a slightly better emulsion stability at pH 6.0 than at pH 2.5. Yet, neither pectin sample was considered having good emulsifying and emulsion-stabilizing properties, indicated by the presence of coalesced and flocculated oil droplets.

## 1. Introduction

Pectin is a complex, biodegradable heteropolysaccharide, which is ubiquitously present in the primary cell wall and middle lamella of higher plants [1]. This polymer plays an important role in plants as it promotes cell–cell adhesion and provides mechanical strength of the cell wall through its interaction with other cell wall compounds [2]. The pectin structure involves three main sub-domains: homogalacturonan (HG), which is considered the linear region of pectin, and two branched domains known as rhamnogalacturonan-I (RG-I), and rhamnogalacturonan-II (RG-II). Pectin can be isolated from different sources, such as legumes [3], fruits and vegetables [4,5], as well as from their waste streams [6]. Particularly, apple pomace is considered the second main source of pectin after citrus peels, encompassing about 14% of the worldwide production of pectin [7,8]. It is mainly produced as a by-product from apple cider and fruit juice production. In general, apple pectin is known to contain, next to HG, considerable amounts of RG-I-related sugars (arabinose, galactose, and rhamnose) within its structure [9,10,11]. Nevertheless, the structural features of apple pectin, and pectin polymers in general, are directed by the nature of the pectin source and the extraction method applied [11,12]. Furthermore, pectin structure modifications can be achieved in a targeted manner by means of enzymatic and/or chemical processes. Until now, pectin modifications have been performed to elucidate the structure of the distinct pectic elements [13,14,15], but also to generate tailored pectin molecules with specific functional properties [16]. In this regard, a considerable number of studies investigated the influence of different pectin structural features on the functional properties of this polymer to provide a deeper insight into the structure–function relationships of pectin [17]. 

In the last decades, an increasing interest in the emulsifying and emulsion-stabilizing (E_F_E_S_) capacity of pectin has been observed. A large number of studies evaluated the effect of specific structural features of pectin, such as the degree of methylesterification [18,19], protein content [20,21], degree of acetylation [19,22,23], ferulic acid content, arabinose and galactose [24], and molecular weight [19,20,25], on the E_F_E_S_ potential of this macromolecule. Some authors focused on investigating the pectin sub-domains and their individual effect on the E_F_E_S_ of pectin to further understand its complex structure–function relation. In this regard, Neckebroeck et al. [26] isolated and studied two different pectin subdomains, HG and RG-I, of carrot pectin. These two domains, as well as the original, non-modified carrot pectin material, were analyzed through different techniques, aiming to gain in-depth insight into the influence of each pectin sub-domain on the E_F_E_S_ capacity of carrot pectin. The authors concluded that a pectin molecule containing both linear and branched regions exhibited the best E_F_E_S_ capacity in comparison to the isolated HG- or RG-rich modified pectin structures. Furthermore, research performed on pectin extracted from okra genotypes [27] suggested that pectin molecules containing RG-I chains of intermediate length, characterized by a molar ratio of (arabinose + galactose)/rhamnose between 2 and 3, exhibited optimum emulsifying properties. Similar research performed by Nakamura et al. [28] supported the positive effect of the RG-I domain on the E_F_E_S_ properties of soybean soluble polysaccharides, attributed to the ramifications (neutral sugar chains) attached to its structure, as well as the ferulic acid–arabinogalactan-protein complexes [24]. Nevertheless, research on the specific effect of the RG-I domain on the E_F_E_S_ potential of apple pectin containing substantial amounts of RG-I within its structure, is limited. 

Additionally, investigating the E_F_E_S_ properties of pectin has been extensively performed on citrus [18,19,29,30,31] and sugar beet pectin [21,32,33,34,35,36], and only limited research can be found in the literature regarding the E_F_E_S_ capacity of pectin from other sources, such as apple pectin [9,37,38,39]. Furthermore, to the best of our knowledge, the E_F_E_S_ potential of structurally modified apple pectin highly rich in RG-I, containing limited amounts of HG, has not been reported yet in the literature; thus, limited information on the relation between such structural characteristics and the E_F_E_S_ of pectin is available. Therefore, in order to better understand the effect of the RG-I domain and the related side chain neutral sugars on the E_F_E_S_ of pectin, structurally different apple pectin-derived materials were generated through a combined enzymatic–chemical approach. In this regard, the present study extracted a RG-I-rich apple pectin-derived compound (AP-RG) by means of an alkaline process from an initial apple pectin material with a DM of 74% (AP74). Furthermore, we investigated the contribution of RG-I-related neutral sugars (arabinose, galactose, and rhamnose) to the E_F_E_S_ potential of the modified apple pectin polymer. This was performed by modifying the side chain neutral sugars present in AP-RG by means of an acid hydrolytic process. Consequently, a second RG-I-rich apple pectin material (AP-RG-hydrolyzed) was generated. The E_F_E_S_ properties of the apple pectin-derived samples in relation to their structural characteristics were assessed through an experimental approach involving the determination of a range of physicochemical and emulsifying properties as well as the physical stability of the emulsions stabilized by one of the three apple pectin-derived samples at two different pH values (2.5 and 6.0). Overall, the results obtained in this work could provide better insight into the contribution of the RG-I domain and side chain neutral sugars to the E_F_E_S_ potential of apple pectin-derived materials.

## 2. Materials and Methods

### 2.1. Materials

Commercial apple pectin (AP) with a degree of methylesterification (DM) of 74% and a molecular weight (M_w_) of 356 ± 28.28 kDa was supplied by Cargill (Belgium) and encoded AP74. The pectin samples were stored in a desiccator until use. Amyloglucosidase from *Aspergillus niger*, α-amylase from *Bacillus licheniformi*, florisil, 3-(*N*-Morpholino)propane sulfonic acid (MOPS), sodium acetate, L-fucose, L-arabinose, D-glucose, D-mannose, and D-xylose were purchased from Sigma Aldrich Chemie GmbH (Steinheim, Germany). D-galactose, sodium nitrate (NaNO_3_), and calcium chloride (CaCl_2_) were purchased from Merck KGaA (Darmstadt, Germany). Sodium hydroxide (1 M), acetic acid, HCl (1 M), and L-rhamnose were obtained from Acros Organics (Geel, Belgium). Fluorescent dye BODIPY FL hydrazide (4,4-difluoro-5,7-dimethyl-4-bora-3a,4adiaza-s-indacene-3-propionylhydrazide) was obtained from Thermo Fisher Scientific (Merelbeke, Belgium). Sodium hydroxide (extra pure flakes) was purchased from VWR Chemicals (Leuven, Belgium). Unless mentioned otherwise, all chemicals used were of analytical grade.

### 2.2. Production of RG-I-Rich Apple Pectin-Derived Samples 

Generation of the RG-I-rich apple pectin-derived samples in this study was accomplished by means of both enzymatic and chemical processes. Given the high amount of glucose (144.05 ± 2.59 mg/g) present in the starting pectin material, residual starch was enzymatically removed using the starch-degrading enzymes amyloglucosidase and α-amylase. Hereafter, β-eliminative processing conditions were applied in order to degrade the HG backbone of AP74, generating an RG-I-rich apple pectin-derived sample (Section 2.2.1). Lastly, the latter pectin sample underwent an acid hydrolysis process to modify the rhamnose, arabinose, and galactose content, generating a hydrolyzed RG-I-rich apple pectin-derived sample (Section 2.2.2). 

#### 2.2.1. Generation of RG-I-Rich Apple Pectin-Derived Samples

##### Enzymatic Degradation of Residual Starch

The structural characterization of the initial apple pectin material, AP74, revealed the presence of large amounts of glucose (144.05 ± 2.59 mg/g). Therefore, prior to the generation of RG-I-rich pectin samples, we aimed to generate a more pure apple pectin, containing mainly pectin-related sugars (galacturonic acid, rhamnose, arabinose, galactose, fucose, and xylose) and only a minor amount of glucose. For this reason, the starch was enzymatically hydrolyzed following the protocol of the Total Starch Assay Kit (A/AMG, Megazyme Inc., Bray, Ireland), with minor modifications. Briefly, apple pectin (7.5% *w*/*v*) was dissolved in 250 mM MOPS buffer (pH 7.0) containing 5mM CaCl_2_, where after α-amylase was added in a concentration of 766 U/g apple pectin. The enzymatic hydrolysis was performed at 85 °C for 20 min under constant stirring at 250 rpm. Once this step was finalized, the solution was immediately cooled to a temperature of 50 °C. Subsequently, sodium acetate buffer (200 mM, pH 4.5) was added to eventually reach a pectin concentration of 3.75% (*w*/*v*), and α-amyloglucosidase at a concentration of 50 U/g pectin was subsequently added. The enzymatic reaction was performed at 50 °C for 30 min under constant stirring at 250 rpm. At the end of the incubation period, the solution was cooled in an ice bath. Afterwards, ethanol was added until reaching a concentration of 80% (*v*/*v*). This was done to recover the starch-free pectin material. Finally, the solution was centrifuged at 9000 rpm for 20 min at 4 °C (J2-HS centrifuge, Beckman, CA, USA). The pellet, containing the purified pectin sample, was dissolved overnight in 1 L demineralized water at room temperature under constant stirring at 250 rpm. 

##### Generation of RG-I-Rich Pectin Samples by Means of a β-Eliminative Reaction

Generation of the RG-I-rich pectin material was achieved by means of a β-eliminative reaction at a high temperature (85 °C) and high pH (12) according to the work of Santiago et al. [40], with minor modifications. 

The pectin material recovered, as described in Section 2.3.1, was heated to 85 °C. Once the required temperature was reached, we adjusted the pH of the solution to a pH value of 12 by gradually adding hot 4M NaOH. The pectin solution was maintained at 85 °C for 4 h under mild stirring. At the end of this period, the pectin solution was cooled to ambient temperature. Subsequently, ethanol was added to the pectin solution until reaching a final concentration of 80% (*v*/*v*). The suspension was mixed for 30 min and thereafter centrifuged at 9000 rpm for 20 min at 4 °C (J2-HS centrifuge, Beckman, CA, USA). The pellet was dissolved in 350 mL of demineralized water. Once dissolved, the mixture was adjusted to pH 3.5 with 0.1 M NaOH, and subsequently dialyzed (Spectra/Por^®^, MWCO = 3.5 kDa) for 48 h. Finally, the dialyzed pectin solution, containing the RG-I-rich pectin material, was freeze dried and stored in a desiccator at ambient temperature until use. This sample is further referred to as apple pectin rich in rhamnogalacturonan-I (AP-RG).

#### 2.2.2. Generation of RG-I-Rich Apple Pectin-Derived Sample with Modified RG-I-Related Sugars by Means of Acid Hydrolysis

The third pectin sample being studied in this work was produced by means of acid hydrolysis of the RG-I-rich sample (AP-RG) based on the procedure described by Santiago et al. [40], with minor modifications. 

The aim of applying this chemical process was to degrade the side chains of the RG-I domain of AP-RG, thus leading to the generation of a pectin sample rich in RG-I but with different amounts of arabinose and galactose. For this, AP-RG was dissolved overnight in demineralized water at a concentration of 1% (*w*/*v*). On the next day, the solution was adjusted to pH 1 using 1 M HCl and immediately placed in a water bath (Memmert, WBU 45, Mariakerke, Belgium) at 80 °C for 2 h under mild stirring. The reaction was stopped by cooling the solution in an ice bath to ambient temperature. Subsequently, ethanol was added until reaching a final concentration of 80% (*v*/*v*). This suspension was mixed for 30 min and thereafter centrifuged at 9000 rpm for 20 min at 4 °C (J2-HS centrifuge, Beckman, CA, USA). The pellet, containing the hydrolyzed RG-I-rich pectin material, was dissolved in 50 mL of demineralized water. The pH of this pectin solution was adjusted to pH 3.5 with 0.1 M NaOH, and subsequently dialyzed (Spectrum Labs, MWCO = 0.1–0.5 kDa) against demineralized water for 48 h. Finally, the RG-I-rich degraded pectin material was freeze dried and stored in a desiccator until use. This sample is further referred to as apple pectin rich in hydrolyzed rhamnogalacturonan-I (AP-RG-hydrolyzed).

### 2.3. Structural Characterization of the Pectin Samples

The initial pectin material AP74 and the two RG-I-rich pectin samples generated (AP-RG and AP-RG-hydrolyzed) were structurally characterized. In general, the galacturonic acid (GalA) content, degree of methylesterification (DM), molecular weight (M_w_) distribution, neutral monosaccharide profile and content, and protein content were determined as described below. 

#### 2.3.1. Galacturonic Acid Content

The galacturonic acid (GalA) content was determined after the samples underwent acid hydrolysis with sulfuric acid (in duplicate) based on the method described by Ahmed and Labavitch [41]. The GalA content was analyzed in triplicate following the spectrophotometric assay described by Blumenkrantz and Asboe-Hansen [42] at a wavelength of 520 nm (GENESYS™ 30 Visible Spectrophotometer, Thermo Scientific, Boston, MA, USA).

#### 2.3.2. Degree of Methyl Esterification

The DM of the pectin samples was determined as described by Kyomugasho et al. [43] by means of Fourier transform infrared (FT-IR) spectroscopy (Shimadzu FTIR-8400S, Tokio, Japan). This method involves the measurement of transmittance at wave numbers between 4000 cm^−1^ and 400 cm^−1^. The absorption intensity (I) of the bands located around 1740 cm^−1^ (carbonyl group stretching) and 1630–1600 cm^−1^ (carboxylate group stretching) were used to generate ratio X (i.e., I_1740_/I_1740+1630_). The calibration curve described by Kyomugasho et al. [43], represented by the equation Y = 136.86*X + 3.987, was used to determine the DM of the pectin samples (Y).

#### 2.3.3. Molecular Weight Distribution

High-performance size-exclusion chromatography (HPSEC) (HPLC unit, Agilent technologies, Diegem, Belgium) equipped with multi-angle laser light scattering (MALLS) (PN3621, Postnova analytics, Landsberg, Germany) and refractive index (RI) detection (Shodex RI-101, Showa Denko K.K., Kawazaki, Japan) was used to measure the molar mass distribution of the pectin samples following the procedure described by Shpigelman et al. [44], with minor modifications. Briefly, pectin samples (0.2% *w*/*v*) were dissolved overnight in a 0.1 M acetic acetate buffer (pH 4.4) containing 0.1 M NaNO_3._ The pectin samples were subsequently filtered (Chromafil^®^ A-45/25, 0.45 mm pore size, Macherey-Nagel Gmbh, Duren, Germany), injected (100 μL), and separated using a series of Waters columns: Ultrahydrogel 250, 1000, and 2000, with exclusion limits of 8 × 10^4^, 4 × 10^6^, and 1 × 10^7^ g/mol, respectively (Waters, Milford, MA, USA). Elution of the pectin polymers was accomplished with a 0.1 M acetate buffer (pH 4.4) containing 0.1 M NaNO_3_ at 35 °C and a flow rate of 0.5 mL/min. The dn/dc value used to calculate the concentration was 0.146 mL/g. Analysis of the samples was performed in duplicate. Calculation of the average molecular weight was achieved by applying the Debye fitting method (2nd order) of the operating software (Nova Mals, version 1.0.0.18, Postnova analytics, Lansberg, Germany).

#### 2.3.4. Neutral Sugar Content

High-performance anion-exchange chromatography (HPAEC) coupled with pulsed amperometric detection (PAD) was used to determine the neutral sugar composition of the pectin samples, and was based on the work of De Roeck et al. [45], with minor modifications. A pectin solution (0.5% *w*/*v*) was mixed with 8 M trifluoroacetic acid (TFA) in a 1:1 ratio, hydrolyzed at 110 °C for 1.5 h, cooled to ambient temperature, and dried using a N_2_-evaporator (Techne FDB03DD, Cambridge, UK) at 45 °C. Afterwards, 1 M ammonium hydroxide was added to neutralize the TFA previously added, and subsequently a second drying step was performed under N_2_ gas at 45 °C. The residue after the second drying step was dissolved in ultrapure water (organic free, 18.2 MΩ cm resistance) and filtered through a 0.45 μm filter (Chromafil^®^ A-45/25, Macherey-Nagel, Duren, Germany) before injection. Analysis of the neutral sugar composition of the hydrolyzed samples was performed in duplicate by HPAEC-PAD [46]. A Dionex system (ICS-6000) equipped with a Dionex Single Pump, a CarboPac™ PA20 column (150 × 3 mm), a guard column (30 × 3 mm), and an ED50 electrochemical detector (Dionex, Sunnyvale, CA, USA) was used. The HPAEC-PAD analysis consisted of an initial equilibration step with 100 mM NaOH for 5 min followed by 4 mM NaOH for 5 min. Subsequently, injection and elution of the hydrolyzed sample (10 μL) was performed isocratically with 4 mM NaOH at a flow rate of 0.5 mL/min for 20 min at 30 °C. Quantification and identification of the different monosaccharides present in the samples was achieved by use of calibration curves of commercial sugar standards (L-fucose, L-rhamnose, L-arabinose, D-galactose, D-glucose, D-xylose, and D-mannose) at varying concentrations (1–10 ppm). In order to correct for possible degradation of the monosaccharides during the acid hydrolysis step, hydrolysis of the sugar standards with TFA (as previously described) was also performed. 

#### 2.3.5. Protein Content

Determination of the total nitrogen content of the pectin samples was performed by means of the Dumas method [47], using an elemental analyzer (Carlo-Erba EA1108 CHNS–O elemental analyzer, Thermo Scientific, Waltham, MA, USA). The total protein content was calculated based on the total nitrogen content determined and applying a correction factor of 6.25. Analysis of each sample was carried out in duplicate. 

### 2.4. Physicochemical Properties of the Pectin Samples

#### 2.4.1. ζ-Potential Measurements

The charge density of the different pectin samples was determined based on the protocol described by Verkempinck et al. [18]. Briefly, the ζ-potential was measured at different pH values by means of automated capillary electrophoresis equipment (Zetasizer NanoZS, Malvern Instruments, Worcestershire, UK). Pectin solutions (0.01% *w*/*v*) were prepared by individually dissolving the pectin material in ultrapure water (organic free, 18.2 MΩ cm resistance) overnight. Subsequently, the pH of each pectin solution was adjusted to a specific pH value, ranging from 1.5 to 8, using 0.1 M NaOH or 1 M HCl. A capillary cell possessing two electrodes on each side was then filled with the pectin solution under analysis. In general, an electric potential is applied over the two electrodes, stimulating particle movement to an extent that depends on the charge density of the particle. The velocity of the hydrocolloid was monitored in order to determine the electrophoretic mobility. Determination of the ζ-potential of the samples was achieved through the software of the system, which applied the equation of Henry. All ζ-potential measurements were performed in duplicate.

#### 2.4.2. Dynamic Interfacial Tension

Changes in the interfacial tension as function of time for the different pectin samples were recorded using a pendant drop tensiometer (CAM 200, KSV Instruments, Helsinki, Finland), as previously described [18]. For this, different individual pectin solutions (0.1 and 1% *w*/*v*) were prepared, using ultrapure water set at pH 2.5 or 6.0. Specifically, the AP-RG and AP-RG-hydrolyzed solutions were prepared at a concentration of 0.1% *w*/*v* for this analysis, due to the turbid color of the solutions at 1% *w*/*v*, which did not allow the visualization of the oil droplet for its analysis. During the 40 min measurement, an oil droplet of purified sunflower oil (pSO) was formed at the tip of a U-shaped needle, located inside a cuvette containing one of the pectin solutions. Purification of commercial sunflower oil was performed with the aim of removing inherently present surface-active compounds. This is necessary to be able to ascribe changes in the interfacial tension to the pectin only. Briefly, a mixture of Florisil and commercial sunflower oil (1:5 ratio) was stirred at 500 rpm for 24 h. The mixture was subsequently filtered and stored in the dark at −40 °C until use. The purified sunflower oil (pSO) was also used during the preparation of the pectin-containing emulsions, described in Section 2.5. Analysis of the interfacial tension for each pectin sample was performed in duplicate.

#### 2.4.3. Flow Behavior 

The viscosity of the different pectins in solution were determined at 25 °C using a stress-controlled rheometer (MCR 302, Anton Paar, Graz, Austria) and double-wall Couette geometry (DG26.7, internal radius 12.3 mm, external radius 13.3 mm, and measuring height 40 mm) as described by Bernaerts et al. [48], with minor modifications. For this purpose, pectin solutions (1% *w*/*v*) were prepared overnight at pH 2.5 or 6.0. Before the actual measurement, an initial pre-sheared step was applied on each solution for 30 s at a rate of 10 s^−1^, followed by 300 s of rest. Thereafter, the flow behavior of the samples was evaluated by applying a logarithmically increasing shear rate from 1 to 100 s^−1^, until reaching a steady state. Each shear rate was performed for a maximum time of 40 s. Reliability of the data was ensured by applying a 0.1 μN torque. The viscosity of each pectin sample was determined in duplicate. 

### 2.5. Preparation of Pectin-Containing Emulsions

Different oil-in-water (*o*/*w*) coarse emulsions were prepared by mixing 5% (*w*/*v*) pSO and 1% (*w*/*v*) pectin solution (pH 2.5 or 6.0) for 10 min at 9500 rpm (Ultra-Turrax T25, IKA, Staufen, Germany). Subsequently, a stabilization step was performed by high-pressure homogenization at 100 MPa (STANSTED SPCH-10, Homogenizing System, Essex, UK) for each coarse emulsion. The stabilized emulsions were further distributed among several test tubes and stored in the dark at −4 °C. A total of six different emulsions were prepared during this work, as the three pectin structures were studied at pH 2.5 and pH 6.0. 

### 2.6. Physical Stability Evaluation of the Pectin-Containing Emulsions

During the storage stability study performed for the different pectin-containing emulsions for 14 days at 4 °C, the physical stability of each emulsion was evaluated. Analyses were performed on Day 0 (the day of emulsion preparation), and then Days 1, 4, 8, and 14. On each analysis day, the particle size distribution, volume-weighted mean particle size (d_4,3_), individual droplet size, and microstructure of each emulsion were evaluated. Additionally, macroscopic pictures were taken each analysis day and an accelerated stability analysis was performed on a freshly prepared emulsion (creaming index). Lastly, pectin was visualized within an emulsion through fluorescent microscopy.

#### 2.6.1. Volume-Weighted Particle Size Distribution

Laser diffraction equipment (Beckman Coulter Inc., LS 13 320, Miami, FL, USA) was used for the analysis of the particle size distribution and mean volume-weighted particle size (d_4,3_) of the pectin-containing emulsions prepared. The emulsions were shaken, brought into a stirring tank filled with demineralized water, and pumped to the measurement cells. The principle of this measurement involves the diffraction of the laser light by the oil droplets present in the sample (wavelength main illumination source: 750 nm; wavelengths halogen light for Polarization Intensity Differential Scattering (PIDS): 450 nm; 600 nm; 900 nm). Analysis of the detected intensity, generated by the diffracted laser light, and its transformation into particle sizes, was attained by applying the Mie model (refractive index). The d_4,3_ values were reported as they are considered more sensitive indicators for emulsion instability. This is related to the susceptibility of the d_4,3_ value to the presence of large particles, even when present in only small amounts. Since each of the emulsions was stored in two different test tubes per analysis day, measurements of particle size distribution and d_4,3_ values were performed in duplicate for each of the tubes containing a particular emulsion. The reported particle size distributions and d_4,3_ values are averages of the four measurements performed for a specific emulsion.

#### 2.6.2. Individual Oil Droplet Size

In order to determine the volume % of oil droplets larger than 1 μm, the Single Particle Optical Sizing (SPOS) technique (AccuSizer 780 APS, Particle Sizing Systems, Santa Barbara, CA, USA) was used. Firstly, a manual dilution step was applied of the original pectin-containing emulsions (1:200) with ultrapure water. After injecting the diluted emulsion into the equipment, a two-stage automated dilution system enables further dilution. This large dilution of the samples is necessary in order to allow the analysis of one oil droplet at a time by maintaining a particle concentration below the preset limit of 3500 particles/mL. All particles passing through the optical-sensing zone of the sensor were counted and sized. A duplicate measurement was performed of each test tube. The reported volume % of oil droplets larger than 1 μm is the average of the four measurements performed. 

#### 2.6.3. Microstructure

Light microscopy (Olympus BX-41 microscope, Olympus, Optical Co. Ltd., Tokyo, Japan) equipped with a XC-50 digital camera, coupled to image-analysis software (cellSens Standard, Olympus, Optical Co. Ltd., Tokyo, Japan), was applied to visualize the microstructure of the different pectin-containing emulsions. 

#### 2.6.4. Fluorescent Microscopy

The microstructure of the different pectin-containing emulsions was additionally visualized through fluorescent microscopy, as described by Kyomugasho et al. [49]. For this analysis, the non-ionic fluorescent dye BODIPY FL hydrazide (4,4-difluoro-5,7-dimethyl-4-bora-3a,4adiaza-s-indacene-3-propionylhydrazide) was utilized to label the pectin samples used for the preparation of the different emulsions. Briefly, generation of covalently labeled pectin materials was performed through the oxidation of the pectin hydroxyl groups to aldehydes, with subsequent covalent linkage of the aldehyde groups to the fluorescent dye. 

Coarse emulsions were prepared by mixing 5% (*w*/*v*) pSO with (1% *w*/*v*) pectin solutions (pH 2.5 or 6.0), with addition of a the fluorescently labelled pectin. Afterwards, the oil–water mixture was mixed for 10 min at 9500 rpm (Ultra-Turrax T25, IKA, Staufen, Germany). An Olympus BX-41 microscope (Olympus, Optical Co. Ltd., Tokyo, Japan) equipped with epifluorescence illumination (X-Cite Fluorescence Illumination, Series 120Q EXFO Europe, Hants, UK) and image-analysis software (CellSens Standard, Olympus, Optical Co. Ltd., Tokyo, Japan) was used to visually analyze each of the coarse emulsions. High-pressure homogenization was not applied, since these larger oil droplets of the coarse emulsion are better visible under the microscope than the rather small ones obtained after high-pressure homogenization. 

#### 2.6.5. Accelerated Physical Stability Test

Evaluation of the physical stability of the pectin-containing emulsions was also achieved using an analytical centrifuge (LUMiFuge, LUM GmbH, Berlin, Germany) as described by Neckebroeck et al. [26], with minor modifications. Specific cuvettes were filled with emulsion up to the indicated line and closed to avoid spillage during analysis. Subsequently, the cuvettes were placed inside the equipment and centrifuged at 20 °C at 1000× *g*. During centrifugation, a transmission profile is recorded every 45 s, which are used to calculate the overall instability index using SEPView^®^ 6 software (LUM GmbH, Berlin, Germany), which is based on Space- and Time-resolved Extinction Profiles (STEP^®^) Technology^®^ [50]. A total of 1000 consecutive transmission profiles were recorded per emulsion. The analyses were performed in duplicate.

### 2.7. Statistical Analysis

The statistical software JMP (JMP Pro15, SAS Institute Inc., Cary, NC, USA) was used for the different statistical analyses. Significant differences were evaluated by means of one-way ANOVA and Tukey’s Studentized Range post-hoc test. The level of significance was 95% (*p* < 0.05).

## 3. Results and Discussion 

This research focused on evaluating the structural modifications of apple pectin and how it affects emulsifying and emulsion-stabilizing properties. In this regard, the results on the structural characterization of the evaluated pectin materials AP74, AP-RG, and AP-RG-hydrolyzed are discussed in detail in Section 3.1. Furthermore, the results obtained regarding the physicochemical/emulsifying properties of the three pectin samples are presented in Section 3.2. Finally, Section 3.3 contains the data obtained by the different methods used to study the physical stability of the *o*/*w* pectin-containing emulsions prepared at two pH conditions (2.5 and 6.0). 

### 3.1. Structural Characterization of Apple Pectin and RG-I-Rich Apple Pectin Samples

Two RG-I-rich pectin samples (AP-RG and AP-RG-hydrolyzed) were generated by structurally modifying the initial apple pectin sample (AP74) with a yield of 30 and 40 wt %, respectively. Since the RG-I-rich pectin samples were produced by structurally modifying AP74, it is expected that the three pectin materials exhibit structural differences among each other. Therefore, AP74, AP-RG, and AP-RG-hydrolyzed were structurally characterized in terms of GalA content, neutral sugar content, DM, M_w_, and protein content. The results obtained are presented and discussed in the following sections. In general, gaining insight into the structural properties of pectin is of major importance to understand the impact on the E_F_E_S_ potential of this complex polymer. 

#### 3.1.1. Galacturonic Acid and Neutral Sugar Content

The structural characterization of the three pectin materials is presented in Table 1. 

Galacturonic acid content: From the results obtained, it can be observed that the GalA content of AP74 (647.39 ± 5.87 mg/g) is significantly (*p* < 0.05) higher than that of AP-RG (197.09 ± 9.32 mg/g) and AP-RG-hydrolyzed (265.69 ± 9.02 mg/g). These results were expected, as AP-RG and AP-RG-hydrolyzed were generated by degrading the HG domain of the initial pectin material AP74. The lower GalA content in the RG-I-rich pectin samples confirms the successful generation of pectin samples containing abundant amounts of RG-I and limited amounts of HG. On the other hand, the GalA content of the AP-RG-hydrolyzed sample was higher than that of the AP-RG sample, indicating a selective removal of side chain neutral sugars during the hydrolysis step.

Neutral sugar content: The neutral sugar content (Table 1) revealed that mannose was present in the lowest amount in the three pectin materials, while glucose and galactose were the most abundant sugars in AP74 and the RG-I-rich pectin samples, respectively. High amounts of glucose have been attributed to the possible simultaneous extraction of pectin, hemicellulose, and starch during the extraction process as side products [51,52]. The low glucose content in AP-RG and AP-RG-hydrolyzed indicates the successful degradation and thus removal of residual glucose-containing polymers present in the starting material, AP74. Furthermore, the results clearly showed that the RG-I-related sugars (arabinose, galactose, and rhamnose) are present in relatively low amounts in AP74, while they are more abundant in both RG-I-rich pectin samples. This indicates the limited contribution of RG-I in the overall structure of the initial AP74 pectin sample. The hydrolysis of the AP-RG sample (leading to the AP-RG-hydrolyzed sample) merely removed arabinose from the side chains. Pentoses, such as arabinose and xylose, are known to hydrolyze faster under acid conditions at high temperatures (>80 °C) [53,54,55].

Sugar ratios: In addition, different sugar ratios (linearity, RG-I contribution, degree of branching, and HG/RG ratio) [46,56] were calculated from the neutral sugar composition of the three pectin materials (Table 1). The results obtained show that AP74 has the highest linearity among all samples evaluated (5.59 ± 0.53%), indicating the high content of HG within its structure. This result is supported by the high HG/RG ratio (Ratio 4) obtained for this sample, being an indicator for linear pectins rich in HG. Furthermore, AP-RG and AP-RG-hydrolyzed exhibited the highest values for Ratio 2 (0.25 ± 0.01 and 0.10 ± 0.01%, respectively), related to the contribution of RG-I to the entire pectin structure. These results confirm that the alkaline treatment of AP74 led to the successful generation of highly pure RG-I-rich pectin samples. In addition, it was shown that AP-RG and AP-RG-hydrolyzed are the pectin materials containing higher amounts of side-chain sugars (Ratio 3) in comparison to AP74, once more being an indicator of the successful isolation of RG-I by means of a β-eliminative reaction during which the side chains of the RG domain were not degraded. Finally, the HG/RG ratio (Ratio 4) for both AP-RG and AP-RG-hydrolyzed (0.31 ± 0.01 and 0.68 ± 0.01%, respectively) are much lower in comparison to the ratio of AP74, indicating the limited presence of HG within these former samples. Insight into the sugar composition of the evaluated pectin samples is particularly important in this research, as the pectin samples exhibited differences in structure, including the side-chain-related sugars (arabinose, galactose, and rhamnose). 

#### 3.1.2. Degree of Methylesterification

From Table 1, it can be seen that AP74 exhibited a high DM of 74%, while the generated RG-I-rich samples were completely demethylesterified (DM < 3%) during the alkaline production process. This was expected as it is known that the methyl-ester groups located at the C-6 position of GalA residues can undergo hydrolysis at near-neutral conditions (pH 5.0) [17]. The hydrolysis reaction rate was reported to increase with increasing temperature. Furthermore, under the aforementioned conditions, β-eliminative degradation of pectin and chemical demethylesterification are able to occur concomitantly. Similar results were reported after isolation of an RG-I-rich pectic material from carrots (5.89 ± 2.51%) [26] and carrot purée (4.4 ± 0.7%) [40]. 

#### 3.1.3. Protein Content

The proteinaceous moiety of pectin facilitates the adsorption of the polymer at the oil–water interface [16]. Therefore, determining the protein content is of great importance, as a protein amount of only 3% could significantly enhance the emulsifying properties of pectin [57]. The three samples evaluated in this work, AP74, AP-RG, and AP-RG-hydrolyzed, presented no significant differences among them (1.52 ± 0.01, 1.41 ± 0.44, and 1.13 ± 0.15%, respectively). This rather low protein content is only expected to play a limited role in the E_F_E_S_ potential of the pectin samples evaluated in this research.

#### 3.1.4. Molecular Weight Distribution

**Figure 1:**
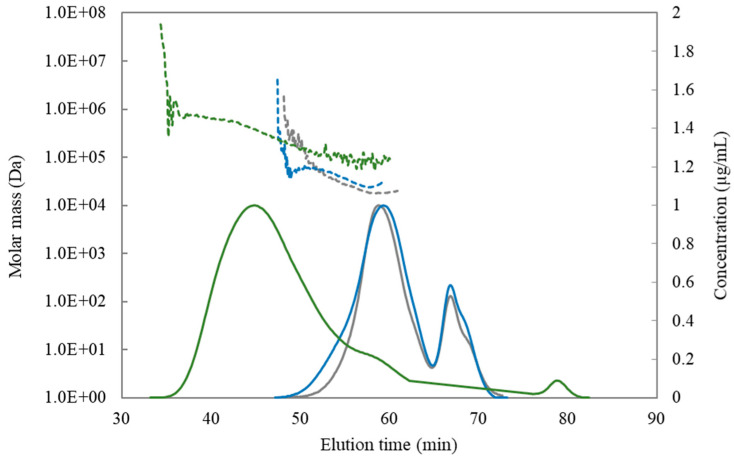
Molecular weight (M_w_) distribution (dashed lines) and concentration profiles (full lines) of AP74 (green), AP-RG (blue), and AP-RG-hydrolyzed (grey).

The M_w_ distribution and concentration profiles of AP74, AP-RG, and AP-RG-hydrolyzed are shown in Figure 1, while their average M_w_ is presented in Table 1. In general, it can be observed that the alkaline process applied led to the production of pectin samples with a lower M_w_ than the original AP74. This is evidenced by a shift in the M_w_ profile (dashed lines) of the RG-I-rich pectin samples towards the right. Besides, smaller differences in M_w_ distribution among both RG-I-rich pectin samples were observed. Although the retention time profiles are rather similar (reflecting the hydrodynamic volume of the polymers), the M_w_ further decreased due the removal of side chain neutral sugars during the acid hydrolysis (from AP-RG to AP-RG-hydrolyzed). Additionally, the concentration profiles (full line) of the three pectin samples exhibited a unimodal shape, indicating a single pectin population. In case of both AP-RG and AP-RG-hydrolyzed, a second concentration peak (eluting at 68 min) was observed. This elution peak indicated the presence of GalA units, resulting from the HG alkaline degradation and impurities (mainly residual salts, e.g., Na^+1^) that were not able to be completely removed during the purification process of the samples (dialysis for 72 h with a MWCO = 0.1–0.5 kDa). Therefore, only the data collected up to 64 min (elution time of mono-galacturonic acid) were considered for the calculation of the average M_w_ of the three pectin samples (Table 1). 

### 3.2. Physicochemical Properties of the Pectin Samples

Besides the analyses performed on the structural characteristics of the three AP-derived samples, particular physicochemical properties were investigated in order to evaluate the E_F_E_S_ potential of these samples. This will allow to correlate the results obtained with the structural characteristics they exhibit. Specifically, the ζ-potential, interfacial tension, and flow properties of the pectin solution were evaluated. The results are described and discussed in detail in the following sections.

#### 3.2.1. ζ-Potential 

**Figure 2:**
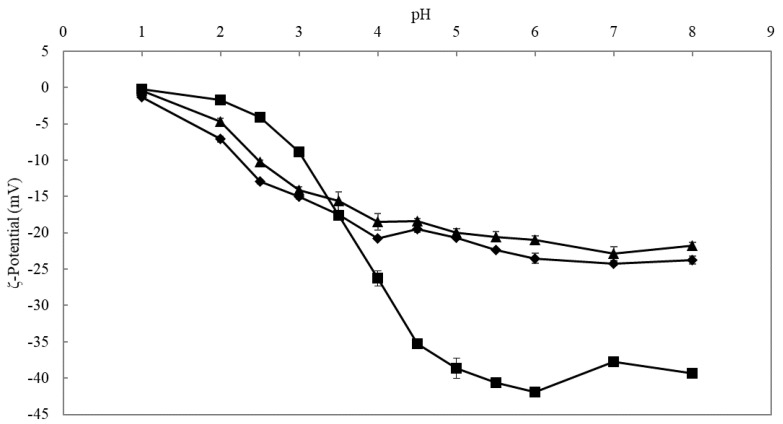
ζ-Potential of the 1% *w*/*v* solutions of the differently generated apple pectin-derived samples (■ AP74, ▲ AP-RG, and ♦ AP-RG-hydrolyzed) as a function of pH.

The free carboxyl groups of the GalA residues, located along the backbone of the pectin molecule, can undergo ionization, meaning that they can carry a negative charge. This structural characteristic can impact the functional properties of pectin. For instance, it was shown in previous research that the DM of the pectin molecule largely impacted its charge density [18,26]. In this regard, the three apple pectin-derived samples generated (i.e., AP74, AP-RG, and AP-RG-hydrolyzed) were analyzed in terms of their ζ-potential at different pH values (Figure 2). 

It could be observed that all pectin samples carried negative charges regardless of pH, which was expected since pectin is known to be an anionic cell wall polysaccharide [58]. At pH values below the pKa of pectin (3.38 to 4.10), the non-modified AP74 sample exhibited a less negative charge density as compared to AP-RG and AP-RG-hydrolyzed. This observation can be explained based on the structural features of AP74. Although AP74 contained the highest GalA content, it also showed a high DM, which means that the amount of free carboxylic groups is relatively low. Therefore, the results indicated that the available carboxylic groups present in AP74 are undissociated at pH values below the pKa of pectin [59,60], thus leading to a less negative charge density in comparison to the two remaining samples, AP-RG and AP-RG-hydrolyzed, under such pH conditions. In contrast, at pH values above the pKa of pectin, AP74 exhibited the most negative charge density among the three samples. This trend was more pronounced as the pH increased. These results are expected, since AP74 contained a relatively high amount of free carboxylic groups as compared to AP-RG and AP-RG-hydrolyzed. The latter two samples only contained a limited amount of HG, and hence had fewer available free carboxylic groups that could be ionized.

#### 3.2.2. Dynamic Interfacial Tension

**Figure 3:**
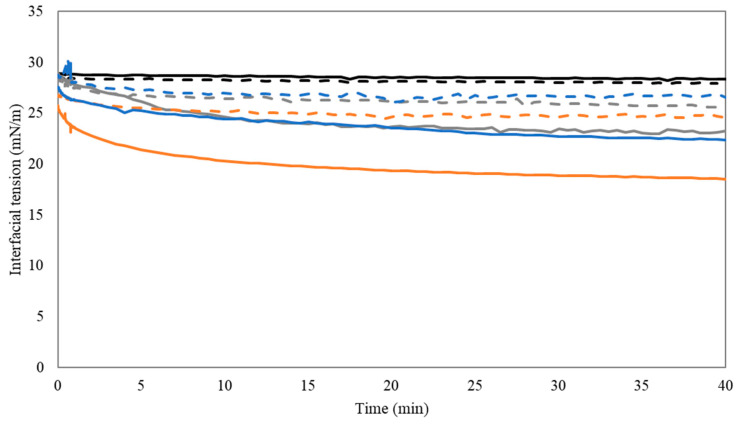
Interfacial tension as a function of time for purified sunflower oil in a 1% solution of AP74 (grey), 0.1% solution of AP-RG (orange), and 0.1% solution of AP-RG-hydrolyzed (blue), at pH 2.5 (full lines) and 6.0 (dashed lines). The interfacial tension of purified sunflower oil in ultrapure water (black) at pH 2.5 and 6.0 was determined for comparison of the results obtained.

A reduction in the interfacial tension of oil droplets present in a aqueous environment is desirable in order to avoid instability of the system by impeding droplet coalescence [61]. Knowledge regarding the interfacial properties of compounds, aimed to be used as emulsifiers, is essential to determine the emulsifying potential they might exhibit. In this regard, the dynamic interfacial tension of purified sunflower oil droplets present in 0.1 and 1% pectin solutions, as a function of time, was determined at pH 2.5 and 6.0. Additionally, determination of the interfacial tension of purified sunflower oil in only ultrapure water was performed under the same pH conditions, as a reference condition. The results obtained are presented in Figure 3. 

From this figure, it can be seen that the interfacial tension of the sunflower oil in ultrapure water did not decrease and was constant at both pH conditions, indicating the absence of emulsifying compounds in the purified sunflower oil. Therefore, the drop in interfacial tension of the sunflower oil droplets created in the different pectin solutions can be attributed to the specific adsorption of the pectin molecules. 

In general, all the pectin samples analyzed decreased the interfacial tension of the sunflower oil droplet to a certain extent at both pH conditions, thus confirming their surface-active character. Generally, this decrease was less pronounced at pH 6.0. The limited decrease in interfacial tension at pH 6.0 for all the pectin materials evaluated, could be explained by the charge density of this pectin material, which might have influenced pectin molecules to experience intermolecular electrostatic repulsions, thus hampering the complete coverage of the oil droplets interface under such conditions.

Furthermore, the results showed that the AP-RG sample at pH 2.5 exhibited the highest potential to decrease the interfacial tension among all samples. This sample exhibited the highest content in RG-I-related sugars among the three samples studied; thus, it is suspected that these sugar molecules mediated the intermolecular associations between the pectin molecules at pH 2.5, leading to the formation of an interfacial layer that led to a decreased interfacial tension. The presence of (RG-I-related) neutral sugar side chains as well as protein moieties can contribute to the stability of emulsions through the formation of hydrated adsorbed layers at the oil–water interface, which prevent emulsion destabilization [36]. 

In addition, upon comparison of both RG-I-rich pectin-containing emulsions (i.e., AP-RG and AP-RG-hydrolyzed), it can be observed that, under acidic conditions, the latter sample performed the least. From the structural characteristics of both pectin samples described in Table 1, it can be observed that AP-RG contained the highest amount of both RG and Ratio 2 (related to the contribution of the RG-I domain) among all the pectin materials. Overall, these results indicate that the abundant amount of the RG domain within this sample structure positively influenced the interfacial properties it exhibited. On the contrary, at pH 6.0, both RG-I-rich samples displayed a relatively comparable behavior; thus, under such pH conditions, no major differences in terms of emulsion stability are expected between both pectin samples.

#### 3.2.3. Determination of Flow Properties

Viscosity is an important property, playing an essential role in the stability of an emulsion. Specifically, the viscosity of the continuous phase is of high relevance as it can influence the short- and long-term stability of an emulsion by reducing the mobility of the oil droplets, thus impeding droplet collision and consequently coalescence [37]. 

**Figure 4:**
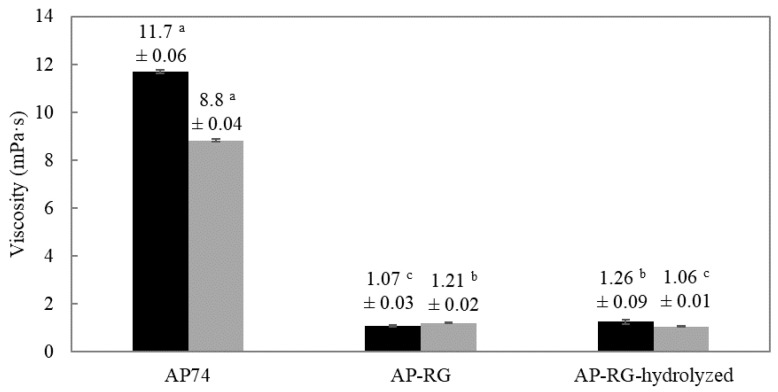
Viscosity of the 1% *w*/*v* AP74, AP-RG, and AP-RG-hydrolyzed solutions at a shear rate of 10 s^−1^. Measurements performed at pH 2.5 are represented by black bars while grey bars represent the results obtained at pH 6.0. Different superscript letters indicate significant differences (*p* < 0.05) among the viscosities of the different pectin materials at pH 2.5 and 6.0, respectively.

In this regard, the viscosity of a 1% *w*/*v* solution of the three apple pectin-derived samples was determined at pH 2.5 and 6.0 using a shear rate of 10^−1^ (results shown in Figure 4). It can be observed that the original pectin AP74 exhibited a significantly higher viscosity in comparison to the RG-I-rich derived samples AP-RG and AP-RG-hydrolyzed. This could be explained by the fact that AP74 did not undergo any structural modification, meaning that it contains both a linear (HG) and a branched (RG) domain within its structure. Therefore, this pectin sample exhibited a higher M_w_ among all the samples investigated, which consequently led to a higher viscosity of AP74 [62]. A high viscosity for a non-modified AP sample was also observed by Neckebroeck et al. [9]. Since both RG-I-rich pectin samples contained considerably lower amounts of HG, higher amounts of RG, and had a significant lower M_w_ (see Table 1) than AP74, interactions between the pectin chains were most probably more limited than for AP74. Consequently, this reduced the overall viscosity of AP-RG and AP-RG-hydrolyzed in comparison to AP74. For the RG-rich samples, relatively similar viscosities were noticed, regardless of pH. It seemed that the further partial hydrolysis of the side chains did not affect viscosity. Once more this might indicate that the E_F_E_S_ properties of these two samples are very similar. Lastly, a slightly increased viscosity of AP74 was observed at pH 2.5 in comparison to pH 6.0. As suggested by Neckebroeck et al. [9], this could be explained by the branched structure and high DM of the AP74 sample. These structural characteristics could hinder the formation of intertwined electrostatic complexes among pectin chains at pH values above the pKa of pectin (around 3.38 to 4.10), thus leading to a lower viscosity of the polymer under such conditions [47].

An increased viscosity of the continuous phase (e.g., due to pectin addition), is known to significantly affect droplet breakup during emulsion preparation upon the application of disruptive forces. Furthermore, the manner in which droplet break-up takes place is directed by the ratio of the viscosity of the dispersed phase (η_D_) to the viscosity of the continuous phase (η_C_) [63]. In this regard, based on extensive research performed on droplet disruption, it has been stated that at viscosity ratios (η_D_/η_C_) below 0.05 or above 5, droplets are resistant to break up, thus negatively affecting the emulsion stability. Therefore, the η_D_/η_C_ ratio of the six 1% *w*/*v* pectin solutions prepared was calculated, and the results obtained are shown in Table 2. The viscosity of sunflower oil (η_D_) was 0.0445 ± 0.0003 (Pa·s) [64].

From the results displayed in Table 2, it can be clearly seen that, at both pH conditions, AP74 is the only pectin sample exhibiting a η_D_/η_C_ between 0.05 and 5. Therefore, based on the results obtained, the oil droplets formed upon droplet break-up during the preparation of the AP74 emulsion were expected to be smaller than the ones present in both AP-RG and AP-RG-hydrolyzed-containing fresh emulsions, since the latter two samples exhibit larger η_D_/η_C_ ratios (>5) regardless of the pH tested. 

**Figure 5:**
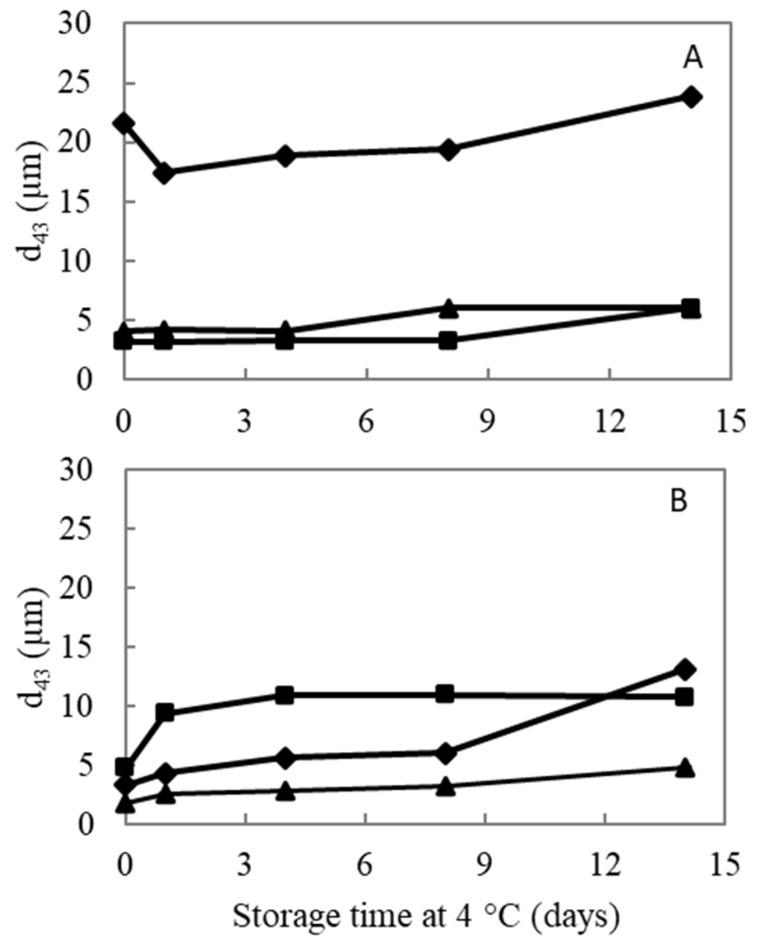
Average oil droplet size (d_4,3_) of the 5% *o*/*w* emulsions stabilized by 1% *w*/*v* (■ AP74, ▲ AP-RG, or ♦ AP-RG-hydrolyzed) at (**A**) pH 2.5 and (**B**) pH 6.0, as a function of storage time under refrigerated conditions (4 °C).

From the results of average particle size obtained and displayed in Table 2 (graphically shown and explained in more detail in Figure 5 and Section 3.3.1 respectively), it can be seen that, as expected, at low pH conditions (2.5), small oil droplets (3.22 ± 0.02 µm) were formed upon preparation of the AP74 emulsion, which can be explained by the low η_D_/η_C_ ratio determined for this pectin sample under acidic conditions. Conversely, at pH 6.0, larger oil droplets were present in the freshly prepared AP74 emulsion in comparison to the emulsions containing AP-RG and AP-RG-hydrolyzed, despite the low η_D_/η_C_ ratio determined for the former sample. These results might be explained by the charge density results obtained for the three pectin samples (Section 3.2.1). It is possible that at pH 6.0, intermolecular repulsion between the negatively charged pectin chains, present in AP74, might play a role, since it was observed that this pectin material exhibited the highest ζ-potential among all the pectin samples investigated at pH conditions above the pKa of pectin. Consequently, it is very plausible that repulsive forces between the pectin molecules, present in AP74, might have hampered the complete coverage of oil droplets at the interface. In this particular case, it can be concluded that the charge density of the pectin material AP74 was the main physicochemical property affecting the initial droplet size upon droplet break-up during emulsion preparation. In the case of AP-RG and AP-RG-hydrolyzed, a combination of interfacial tension properties and structural characteristics of both pectin materials might explain the initial droplet size results obtained for both RG-I-rich pectin samples at pH 6.0, which are discussed in more detail in Section 3.3.1.

### 3.3. Physical Stability Evaluation of the Pectin-Containing Emulsions 

In order to further evaluate the E_F_E_S_ potential of the different AP-derived samples, the physical stability of the 5% oil-in-water emulsions, at both pH 2.5 and 6.0 and stabilized by the diverse pectin samples, was assessed by performing a physical stability study for a period of 14 days at 4 °C. An integrated analytical approach was used, including evaluation of the particle size distribution, average volume-based particle size (d_4,3_), volume % of oil droplets larger than 1 μm, microstructure (light and fluorescence microscopy), macroscopic pictures, and instability index.

#### 3.3.1. Particle Size and Microscopic Assessment

**Figure 6:**
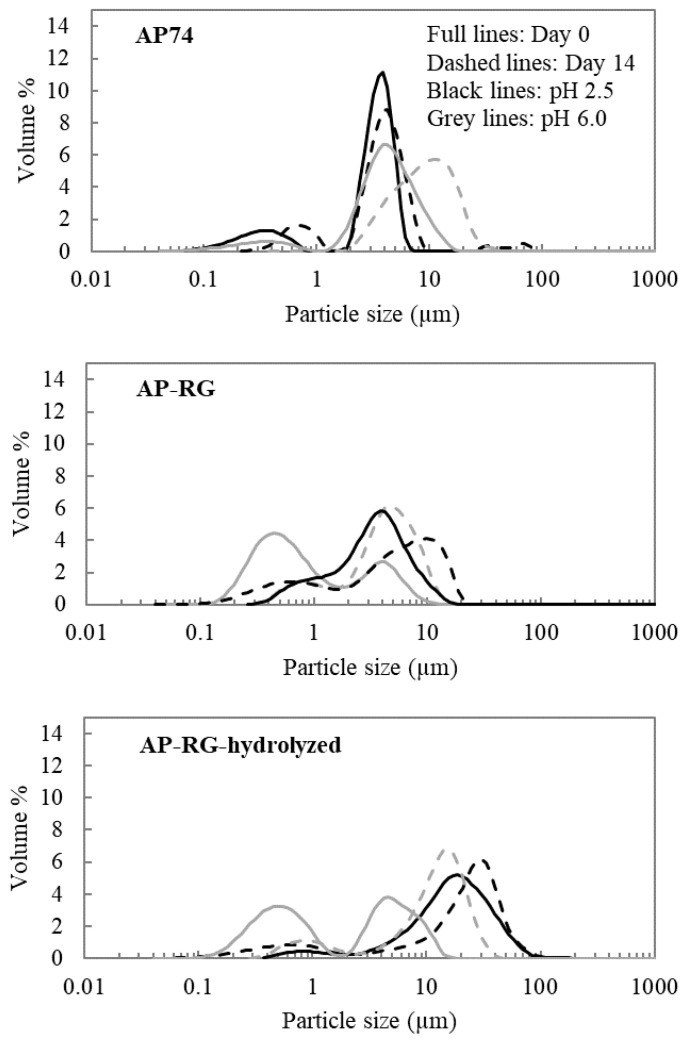
Particle size distribution of each pectin-based emulsion, determined on the day of emulsion preparation (full line) and at the end of the storage period (Day 14) (dashed line) at 4 °C. Black lines represent the emulsions at pH 2.5 and grey lines represent the emulsions at pH 6.0.

**Figure 7:**
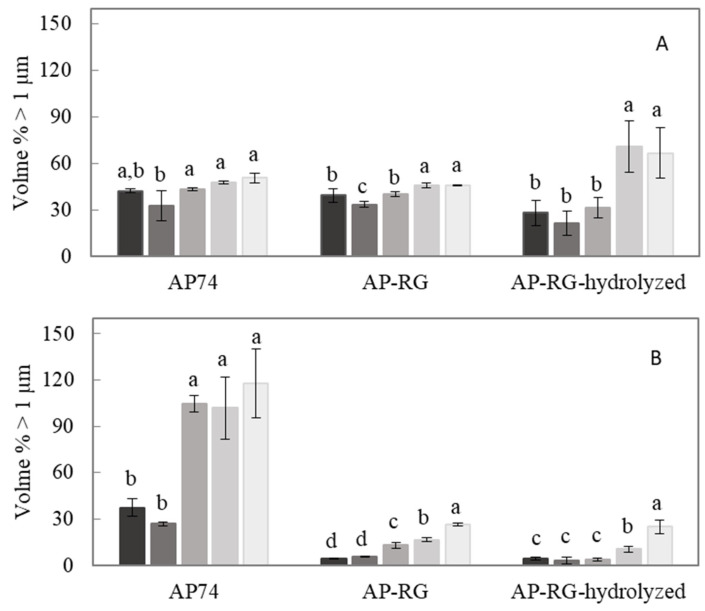
Volume fraction of oil droplets larger than 1 μm present in the 5% *o*/*w* emulsions stabilized by 1% *w*/*v* AP74, AP-RG, or AP-RG-hydrolyzed at (**A**) pH 2.5 and (**B**) pH 6.0. Bars with increasing color intensity represent the volume % of large droplets determined on ■ Day 0, ■ Day 1, ■ Day 4, ■ Day 8, and ■ Day 14. Different lowercase letters indicate significant differences (*p* < 0.05) in the volume fraction of oil droplets larger than 1 μm, determined on different storage days (0, 1, 4, 8, and 14), for each pectin sample.

**Figure 8:**
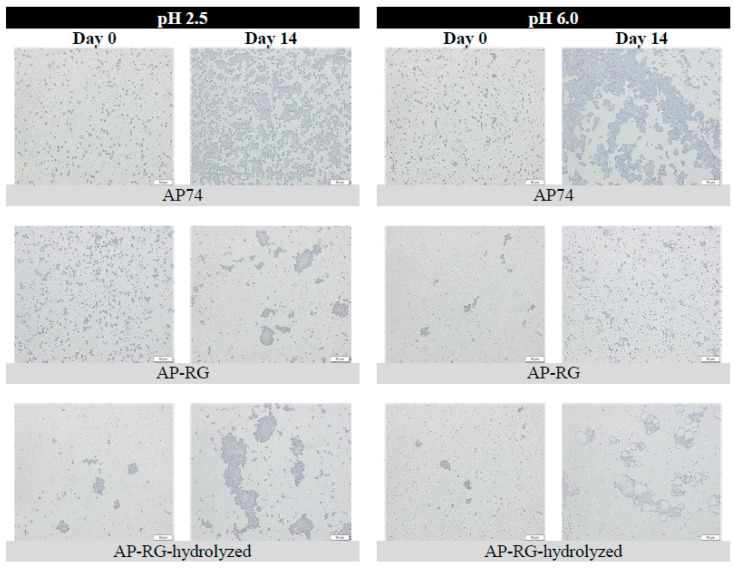
Microscopic images of the 5% *o*/*w* pectin-based emulsions stabilized by the different (1% *w*/*v*) pectin samples in solution at pH 2.5 and 6.0. Scale bars represent a length of 50 μm.

Evaluation of the oil droplet size present in the emulsified system was determined by analyzing the particle size distribution (PSD) as well as the volume-weighted average particle size (d_4,3_), which were determined on different days (Days 0, 1, 4, 8, and 14) during the entire storage period. The average d_4,3_ values obtained on each day of analysis during the storage period are depicted in Figure 5. Additionally, Figure 6 shows the PSD of the emulsions stabilized by AP74, AP-RG, or AP-RG-hydrolyzed, both at pH 2.5 and 6.0, at the day of emulsion preparation (Day 0), and on the last day of the storage period (Day 14). Additionally, determining the volume % of particles larger than 1 μm (Figure 7) was assessed by means of Single Particle Optical Sizing (SPOS). This technique allows the quantification of single oil droplets. Finally, microscopic pictures of each emulsion were taken to visually assess oil droplet size and for the identification of destabilizing mechanisms, such as flocculation and coalescence, which might be taking place as a function of storage time (Figure 8). 

AP74 sample: It can be clearly seen that the PSD of AP74 shifted towards the right after 14 days of storage (Figure 6A), indicating emulsion destabilization. This was observed at both pH conditions (2.5 and 6.0), yet being more pronounced at pH 6.0. Besides, the microscopic pictures revealed the presence of floccules from the day of emulsion preparation at both pH conditions. Therefore, it is suggested that the PSD of these AP74 emulsions encompassed single oil droplets as well as flocculated ones. An increasing amount of larger droplets (larger than 1 μm) was observed during the 14 days of storage at pH 6.0 (Figure 7), pointing to the coalescence phenomenon. On the contrary, at pH 2.5, the individual oil droplet size remained constant, thus indicating that flocculation was the predominant destabilization phenomenon taking place. The improved stabilization of AP74 at pH 2.5 is suggested to be mainly attributed to the low viscosity ratio (between the oil phase and the continuous phase) determined for AP74 at pH 2.5, which enabled the formation of small initial oil droplets within the dispersed system. Another plausible explanation for the improved emulsifying potential of this pectin material under acidic conditions is related to the formation of a denser layer at the surface of the oil droplets, owed to reduced intramolecular repulsions taking place at low pH conditions. Therefore, impeding destabilization mechanisms, such as flocculation and coalescence, are taking place [16]. As proposed by Verkempinck et al. [18], the lower stability of pectin-containing emulsions at high pH (pH > pKa of pectin) can be ascribed to the more extended adsorption of the complex pectin molecules at the oil–water interface, meaning that the oil droplets are not fully covered by pectin material under such conditions. In other words, the oil droplets present in such systems exhibit a more exposed interface. Furthermore, at high pH, intermolecular repulsions between the negatively charged pectin molecules may hinder the complete coverage of the oil droplets, thus promoting the formation of larger oil droplets [9]. 

RG-I-rich samples: From the day of the emulsion preparation (Day 0), a broad distribution was detected for both AP-RG and AP-RG-hydrolyzed emulsions at pH 2.5 (Figure 6). In addition, both emulsions were characterized by large initial average oil droplet sizes (4.06 ± 0.32 and 23.87 ± 1.39 μm for AP-RG and AP-RG-hydrolyzed, respectively) (Figure 5). Moreover, the micrographs (Figure 8) confirmed the presence of large floccules in both emulsions at a low pH from the day of emulsion preparation. Furthermore, based on the data of the SPOS technique (Figure 7), the presence of relatively large oil droplets was identified in the AP-RG emulsion at pH 2.5 from Day 0; yet, in an amount that remained rather constant during the entire storage period. Oppositely, the AP-RG-hydrolyzed emulsion, which also contained large oil droplets from Day 0, presented increased amounts of these droplets by the end of the storage period. In summary, these results indicate the presence of both coalesced and flocculated oil droplets in both emulsions (AP-RG and AP-RG-hydrolyzed) under acidic conditions from the day of preparation. This poor stability might be explained by the high RG-I content within their structure. It has been proposed that highly branched pectin molecules (i.e., rich in RG-I) exhibit an increased flexibility. This characteristic impedes the formation of a highly interconnected network at the surface of the oil droplet due to the fast desorption of pectin from the interface [27]. Consequently, the space between oil droplets, stabilized by highly flexible pectin chains, are considered to be too narrow, resulting in the inefficient steric stabilization of the dispersed system. Conversely, at pH 6.0, both AP-RG and AP-RG-hydrolyzed emulsions presented a better emulsion stability than at pH 2.5. Although the distributions of both AP-RG and AP-RG-hydrolyzed emulsions exhibited a bimodal shape, the average initial droplet sizes were significantly lower (1.76 ± 0.20 and 3.34 ± 0.35, respectively) in comparison to the respective emulsions at pH 2.5. The volume % of oil droplets larger than 1 μm present in AP-RG and AP-RG-hydrolyzed emulsions was substantially lower in comparison to the results obtained at pH 2.5. The micrographs obtained, shown in Figure 8, revealed the presence of floccules in both emulsions, yet of smaller size than the ones observed at pH 2.5. Nevertheless, these emulsions were also destabilized by the end of the storage period. 

AP-RG versus AP-RG-hydrolyzed: Comparing the results of AP-RG versus AP-RG-hydrolyzed stabilized emulsions revealed that the former emulsion was less unstable than the latter, although both emulsions are unstable at both pH conditions by the end of the storage period. The differences in the results obtained for both emulsions could be linked to the structural differences they exhibit. Based on the saccharide content of both RG-I-rich samples (Table 1), it can be seen that AP-RG contained a lower GalA (19.71 ± 0.93%), higher rhamnose and arabinose (4.14 ± 0.11 and 18.67 ± 0.05%, respectively), and a lower galactose content (22.64 ± 0.01%) in comparison to AP-RG-hydrolyzed (26.57 ± 0.90, 2.29 ± 0.13, 1.79 ± 0.07 and 29.27 ± 0.14%, respectively). In general, the positive impact of the side chains on the E_F_E_S_ capacity of pectin have mainly been attributed to (i) the formation of thick interfacial layers, providing steric stabilization of the dispersed system; and (ii) the interfacial activity of the protein moieties [36,65]. Since the RG-I-rich pectin materials contained only little amounts of protein (ranging from 0.98 to 1.85%), it is less probable that this property had a significant impact on the emulsifying characteristics of these pectin-derived samples. Therefore, it is possible that the larger amounts of RG-I-related sugars in AP-RG slightly improved its E_F_E_S_ properties by forming a more stable and thicker interfacial layer of entangled pectin chains than AP-RG-hydrolyzed. Additionally, it was previously observed (Section 3.2.2) that AP-RG exhibited improved interfacial tension properties in comparison to both AP74 and AP-RG-hydrolyzed, despite the pH; thus, it is very plausible that this physicochemical property might have contributed to the improved stability of both AP-RG emulsions in comparison to the emulsion stabilized by AP-RG-hydrolyzed. Furthermore, the viscosity of both AP-RG and AP-RG-hydrolyzed emulsions exhibited little differences at both pH 2.5 and 6.0, despite their differences in M_w_. In this way, it is assumed that the differences both emulsions exhibit in terms of particle size characteristics are only to a minor extent influenced by the viscosity of the continuous phase.

Based on the structural characteristics of the three pectin samples studied, the emulsion stabilized by the original pectin (AP74) exhibited a better stability than the emulsions stabilized by AP-RG and AP-RG-hydrolyzed. This was most probably related the presence of both linear and branched regions within the structure of AP74. Besides, Wang et al. [30] stated that the equilibrium of the hydrophobicity (mainly related to the content of methyl groups) and hydrophilicity (mainly attributed to the carboxyl and hydroxyl groups) of a pectin molecule highly impacts its emulsifying properties. Therefore, we can consider that this equilibrium is unbalanced for both RG-I-rich pectin samples generated, which negatively affected emulsion stability regardless of pH. Additionally, the relatively high viscosity of AP74 in solution—attributed to its high M_w_—positively affected the viscosity of the continuous phase of the dispersed system, most probably suppressing the mobility of the oil droplets, and so leading to a higher overall emulsion stability.

#### 3.3.2. Microscopic Visualization of Fluorescently Labelled Pectin Molecules in Coarse Emulsions

**Figure 9:**
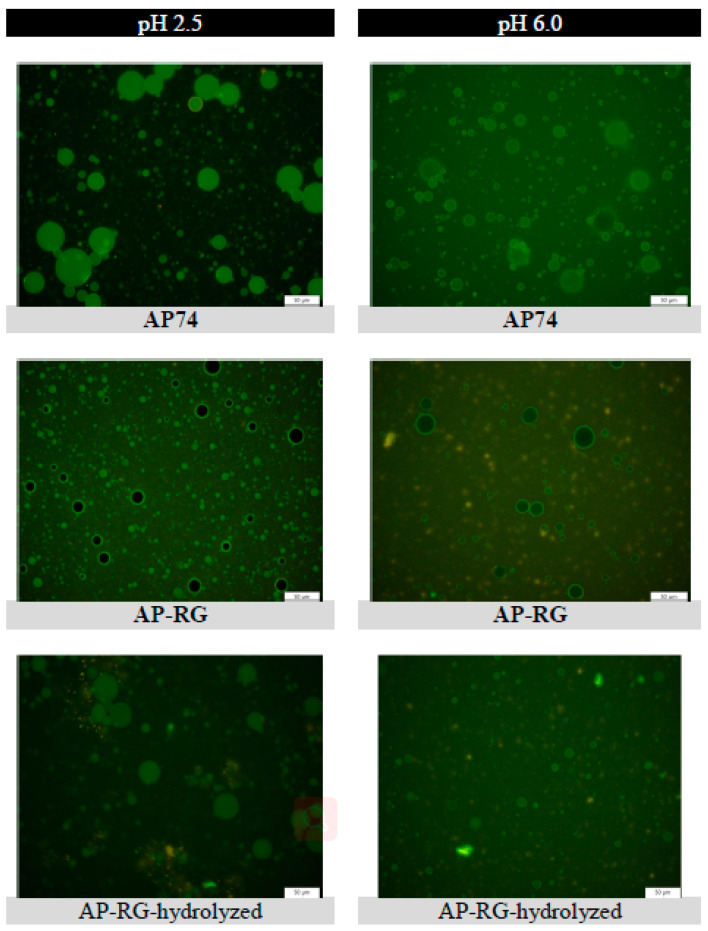
Microscopic images of the different 5% o/w emulsions stabilized by 1% *w*/*v* fluorescently labelled pectin molecules (AP74, AP-RG, or AP-RG-hydrolyzed). The six different microscopic images were captured under the same light intensity and magnification. The scale bar represents a length of 50 μm.

Microscopic pictures of the six emulsions stabilized by labelled apple pectin-derived samples (labelled AP74, AP-RG, or AP-RG-hydrolyzed) were taken on the day of emulsion preparation (Day 0). It was intended to visually identify the location of the pectin compounds studied in this research within the dispersed systems as well as to observe if there were differences in their organization at different pH conditions of the surrounding medium. The micrographs taken are shown in Figure 9. 

In case of AP74, the micrograph at pH 2.5 revealed the presence of the pectin molecules at the oil/water interface, represented by the green-colored oil droplets. The micrograph also exhibited a slightly green fluorescent background, which represented the pectin molecules present in the continuous phase of the dispersed system. However, the color intensity of the background was considerably less than the color intensity of oil droplet surfaces, indicating that the pectin polymers were mainly located at the oil/water interface. On the contrary, at higher pH conditions (6.0), a more intense fluorescent color of the background was visualized than at pH 2.5. The strong fluorescent color of the background indicated an increased amount of labelled pectin molecules in the continuous phase. Combining the results discussed in Section 3.2.1 and these fluorescent micrographs, it can be confirmed that the pectin molecules did not strongly adsorb at the oil droplet interface, thus destabilization of the dispersed system took place through the formation of large oil droplets. In other words, the emulsion at pH 6.0 was less stable than that at pH 2.5. As previously mentioned, the low stability of the emulsions at a high pH is most probably related to the intermolecular repulsions between the negatively charged pectin molecules, which consequently exhibited a more extended adsorption at the oil/water interface, leading to a more exposed interface of the oil droplets.

For the cases of AP-RG and AP-RG-hydrolyzed at both pH 2.5 and 6.0, the micrographs revealed the presence of pectin structures at the surface of the oil droplets as well as in the continuous phase of the dispersed systems. This is visually represented by the green-colored oil droplets surfaces and green-colored backgrounds, respectively. For both RG-I-rich pectin samples, at pH 2.5, a stronger fluorescent color was detected for the background in comparison to the AP74-stabilized emulsion. This indicates the predominant presence of pectin molecules within the continuous phase of the RG-I-rich pectin emulsions. These observation are in agreement with the discussion in Section 3.3.1. On the contrary, at pH 6.0, the micrographs of both RG-I-rich pectin emulsions exhibited a background with a reduced color intensity compared the one of AP74. This confirms that the pectin molecules where located to a higher extent in the continuous phase for the AP74 emulsion at pH 6.0. 

#### 3.3.3. Accelerated Physical Stability Test

**Figure 10:**
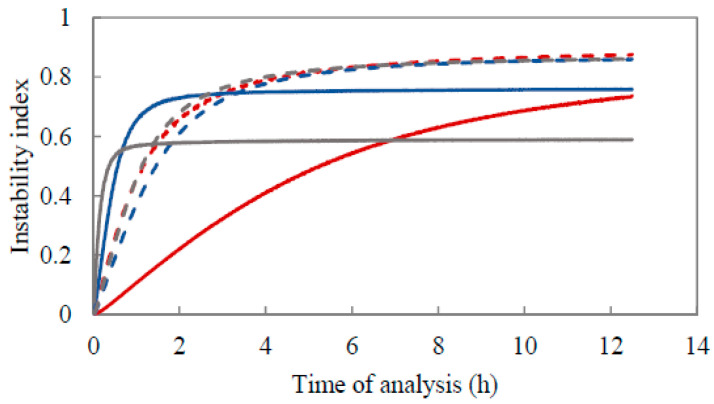
Instability index as function of analysis time for the 5% *o*/*w* emulsions stabilized by 1% (*w*/*v*) AP74 (red), AP-RG (blue), and AP-RG-hydrolyzed (grey) at pH 2.5 (full lines) and 6.0 (dashed lines), respectively.

The physical stability of the three pectin-containing emulsions was quantitatively evaluated through an accelerated test using an analytical centrifuge. In essence, the extent of clarification in the emulsions caused by the applied centrifugal forces is determined through the calculation of the instability index. In Figure 10, the instability index of the six emulsions is displayed as a function of analysis time. A rapid and steep increase in the instability index upon experiencing gravitational stress indicates fast creaming of the emulsion analyzed. 

**Figure 11:**
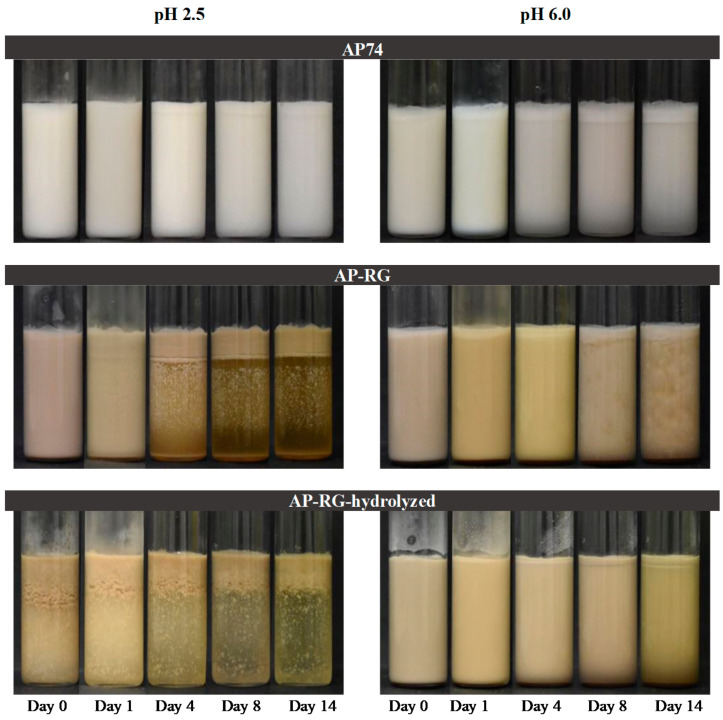
Macroscopic images of the 5% *o*/*w* emulsions, stabilized by AP74, AP-RG, and AP-RG-hydrolyzed, stored at 4 °C. The macroscopic pictures were taken on the day of emulsion preparation (Day 0), and then at Days 1, 4, 8, and 14.

In general, the emulsion stabilized by AP74 at pH 2.5 exhibited the slowest increase in the instability index as function of analysis time among the six emulsions analyzed. This result indicates that this emulsion was more resistant to creaming in comparison to the remaining five emulsions. This observation is in line with the data discussed in Section 3.3.1. These results were also confirmed by the macroscopic pictures displayed in Figure 11, where it can be seen that both AP74 emulsions presented only a thin creaming layer by Day 4, which remained constant over the entire storage period. However, at pH 6.0, the cream layer is slightly ticker, indicating that creaming of this emulsion took place at a faster rate than at pH 2.5. 

Furthermore, when comparing the instability index of the AP74-stabilized emulsion to the results obtained for the emulsions stabilized by AP-RG and AP-RG-hydrolyzed at pH 2.5, it can be seen that both latter samples creamed at a much faster rate. The creaming rate of an emulsion is well known to be directly influenced by the size of the droplets dispersed in the continuous phase of the system [66,67]. Therefore, based on the results described in Section 3.3.1, it is suggested that the evolution of the instability index for the emulsions stabilized by AP-RG or AP-RG-hydrolyzed is indeed greatly influenced by the larger initial size of the dispersed oil droplets present in these emulsions. Furthermore, the presence of non-adsorbed pectin molecules in the continuous phase did not positively affect emulsion stability as the RG-I-rich pectin structures exhibited a relatively low M_w_ (Table 1). These results were confirmed through the macrographs of these emulsions shown in Figure 11. Clear phase separation was observed by the end of the stability study, most probably due to flocculation.

Lastly, when comparing the results obtained at pH 6.0, it can be seen that the three emulsions evaluated exhibited a similar evolution of the instability index as function of analysis time, despite their differences in oil droplet size. These results imply that creaming of the emulsions at a high pH took place at a similar rate and extent. Furthermore, the evolution of the instability indices of these three pectin-containing emulsions indicated that creaming of these emulsions at pH 6.0 took place at a slower rate in comparison to the creaming of both the AP-RG and AP-RG-hydrolyzed emulsions at pH 2.5, but at a faster rate than AP74 at pH 2.5. The macroscopic pictures (Figure 11) confirmed these observations, as from the beginning of the storage period (Day 0) the formation of a cream layer was visual. By the end of the stability study, the cream layer in these three emulsions exhibited a relatively similar thickness, suggesting that creaming of these emulsions took place at a similar rate and to a similar extent after 14 days of storage. 

## 4. Conclusions

This study focused on the role of the RG-I domain in the overall stability of emulsions. For this, three apple pectin-derived samples were prepared, exhibiting structural differences regarding their GalA content, RG-I-related sugars, DM, and M_w_. A highly pure RG-I-rich apple pectin material (AP-RG) was successfully obtained by means of an alkaline treatment of the mother apple pectin. Further acid hydrolysis of AP-RG enabled the extensive removal of arabinose while largely maintaining galactose in the side chains. 

Based on the physicochemical characteristics of these three samples, it was observed that all of them exhibited negative charge densities. However, AP74 exhibited a more negative charge density than the two RG-I-rich samples, ascribed to the high amounts of the HG domain present within its structure, as well as to the high content of free carboxylic groups. The three pectin samples successfully decreased the interfacial tension of a purified sunflower oil droplet at both pH 2.5 and 6.0. Interestingly, AP-RG at pH 2.5 clearly exhibited the highest potential to decrease the interfacial tension among all samples. This was suggested to be attributed to the high amounts of RG-I-related sugars, specifically arabinose, through which a hydrated adsorbed layer at the oil–water interface could be formed. The viscosity of AP74 at pH 2.5 was observed to be the highest among the three pectin samples analyzed, which was ascribed to the presence of both a linear (HG) and branched (RG) domain (leading to a high molecular weight), as this sample did not undergo any structural modifications. 

Furthermore, the storage stability study of the three apple-derived pectin samples revealed the enhanced stability of the non-degraded apple pectin (AP74) at pH 2.5. The formation of small initial oil droplets was mainly attributed to the viscosity ratio (between the oil phase and the continuous phase) and the formation of a denser, more compact layer at the interface, due to the reduced intramolecular repulsions taking place at low pH conditions. Additionally, this emulsion exhibited the highest resistance to creaming, which was ascribed to the improved viscosity-enhancing effect of AP74, through which oil droplet motion was suppressed, retarding the creaming of the emulsion. On the contrary, both RG-I-rich samples exhibited poor stabilizing properties at both pH conditions in comparison to AP74. In addition, differences in arabinose, galactose, and rhamnose between both RG-I-rich pectin samples did not exhibit a significant impact on their emulsifying properties. Overall, coalescence and flocculation were the main destabilizing mechanism observed in the emulsion stabilized by AP74 at pH 6.0, as well as in both RG-rich pectin-based emulsions at pH 2.5. In conclusion, the results obtained in this study suggest that pectin compounds containing an abundant amount of a solely pectin sub-domain (in this case RG-I) perform poorly in comparison to a pectin polymer containing the different subdomains (HG and RG) within its structure. However, differences in the viscosity-enhancing effect among the analyzed samples might be the main factor influencing the stability of pectin-containing emulsions. Therefore, to exclusively understand the specific role of RG-I in the E_F_E_S_ of apple pectin, the viscosity of the pectin-containing emulsions to be evaluated should be standardized. In this way, it would be possible to directly correlate the E_F_E_S_ potential of the pectin polymers exhibiting differences in structure.

## Figures and Tables

**Table 1:** Structural properties of AP74, AP-RG, and AP-RG-hydrolyzed, including the GalA content, neutral sugar content, composition ratios, content of pectin sub-domains HG and RG, protein content, DM, and average M_w_. Results are expressed as average values ± standard deviation.

Property	Samples		
Pectin-Related Sugars (% *w*/*w*)	AP74	AP-RG	AP-RG-Hydrolyzed
GalA	64.74 ^a^ ± 0.59	19.71 ^c^ ± 0.93	26.57 ^b^ ± 0.90
Fucose	0.16 ^a^ ± 0.03	ND	ND
Rhamnose	1.06 ^c^ ± 0.06	4.14 ^a^ ± 0.11	2.29 ^b^ ± 0.13
Arabinose	3.89 ^b^ ± 0.23	18.67 ^a^ ± 0.05	1.79 ^c^ ± 0.07
Galactose	4.42 ^c^ ± 0.71	22.64 ^b^ ± 0.01	29.27 ^a^ ± 0.14
Xylose	0.30 ^c^ ± 0.12	1.04 ^b^ ± 0.01	1.53 ^a^ ± 0.03
**Other sugars**			
Glucose	14.41 ^a^ ± 0.26	0.66 ^b^ ± 0.01	1.00 ^b^ ± 0.05
Mannose	0.05 ^b^ ± 0.03	0.48 ^a^ ± 0.02	0.53 ^a^ ± 0.06
**Pectin domains (% *w*/*w*)**			
HG (GalA-Rha)	63.68 ^a^ ± 0.06	15.57 ^c^ ± 0.11	24.28 ^b^ ± 0.13
RG (2Rha+Ara+Gal)	10.42 ^c^ ± 0.81	49.59 ^a^ ± 0.17	35.63 ^b^ ± 0.18
**Sugar ratios**			
Linearity (GalA/Fuc+Rha+Ara+Gal+Xyl) (Ratio 1)	5.59 ^a^ ± 0.53	0.35 ^b^ ± 0.01	0.69 ^b^ ± 0.01
RG-I contribution (Rha/GalA) (Ratio 2)	0.02 ^c^ ± 0.01	0.25 ^a^ ± 0.01	0.10 ^b^ ± 0.01
Branching of RG-I (Ara+Gal/Rha) (Ratio 3)	7.88 ^b^ ± 1.30	9.91 ^a, b^ ± 0.26	12.53 ^a^ ± 0.72
HG/RG (Ratio 4)	6.12 ^a^ ± 0.47	0.31 ^b^ ± 0.01	0.68 ^b^ ± 0.01
**M_w_ (kDa)**	356.00 ^a^ ± 28.28	49.55 ^b^ ± 0.35	21.55 ^b^ ± 0.07
**Protein content (% *w*/*w*)**	1.52 ^a^ ± 0.01	1.41 ^a^ ± 0.44	1.13 ^a^ ± 0.15
**DM (%)**	73.66 ^a^ ± 2.24	<3%	<3%

Different superscript letters indicate significant differences (*p* < 0.05) among the values of a structural property for the different pectin samples. ND: non-detected. Linearity: high values suggest the analyzed pectin is more linear and less branched. RG-I contribution: small values suggest the analyzed pectin is more linear and less branched. Branching of RG-I: high values suggest the analyzed pectin is highly branched. HG/RG: high values suggest the analyzed pectin is rich in HG.

**Table 2:** Average particle size of freshly prepared pectin-based emulsions, and ratio of the viscosity of purified sunflower oil to the viscosity of pectin in solution. Different superscript letters indicate significant differences (*p* < 0.05) among the values of the average particle size and viscosity ratio, respectively, for the three different pectin samples at a specific pH.

Sample Code	Average Particle Size of Freshly Prepared Emulsions (µm)		η_sunflowewr oil_/η_1% *w*/*v* pectin solution_	
	pH 2.5	pH 6.0	pH 2.5	pH 6.0
AP74	3.22 ^b^ ± 0.02	4.78 ^a^ ± 0.05	3.80 ^b^ ± 0.0001	5.05 ^c^ ± 0.0001
AP-RG	4.06 ^b^ ± 0.32	1.76 ^c^ ± 0.20	41.69 ^a^ ± 0.0001	36.87 ^b^ ± 0.0001
AP-RG-hydrolyzed	21.57 ^a^ ± 1.43	3.34 ^b^ ± 0.35	35.87 ^a^ ± 0.0001	42.03 ^a^ ± 0.0001
